# Supplementary material for: Predicting visual function by interpreting a neuronal wiring diagram
Source: Nature. 2024 Oct 2;634(8032):113–23. doi: 10.1038/s41586-024-07953-5 (PMC11446822; doi:10.1038/s41586-024-07953-5)
Supplement: Supplementary file 6 — CRF and ERF predictions for individual TmY4 and TmY9 cells. Analogous to Supplementary Data 3, but for TmY target types. Shown are the top four monosynaptic pathways, the strongest pathway passing through each of the top ten intermediary types (ranking from Extended Data Fig. 7), and the trisynaptic pathway Tm1–TmY–Dm3–TmY (see the section entitled Prediction of spatial normalization). [file 41586_2024_7953_MOESM6_ESM.zip › DataS4/TmY9q/720575940636880361.pdf]

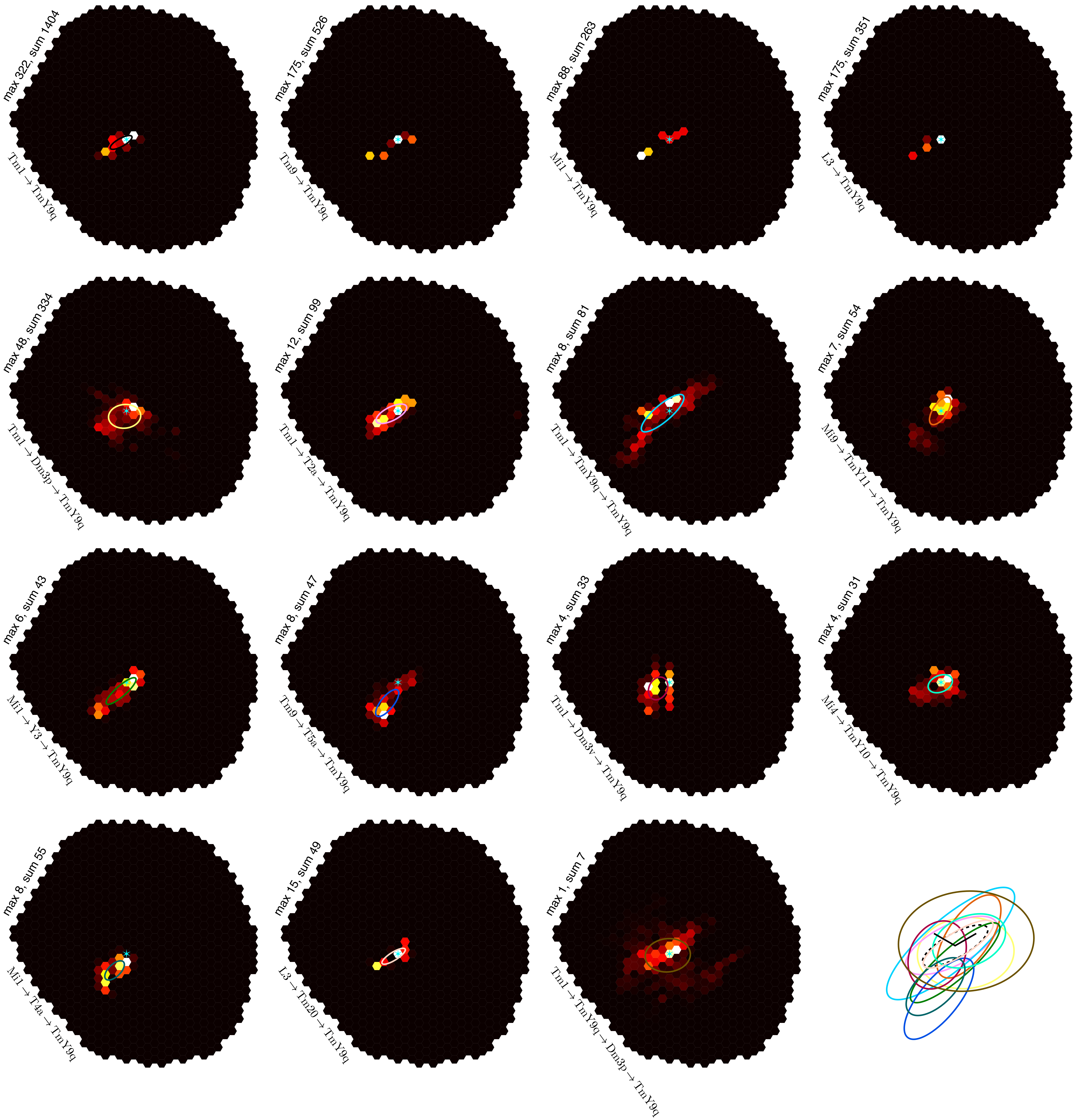

max 322, sum 1404
Tm1 → TmY9q
max 175, sum 526
Tm9 → TmY9q
max 88, sum 263
Mi1 → TmY9q
max 175, sum 351
L3 → TmY9q
max 48, sum 334
Tm1 → Dm3p → TmY9q
max 12, sum 99
Tm1 → T2a → TmY9q
max 8, sum 81
Tm1 → TmY9q → TmY9q
max 7, sum 54
Mi9 → TmY11 → TmY9q
max 6, sum 43
Mi1 → Y3 → TmY9q
max 8, sum 47
Tm9 → T5a → TmY9q
max 4, sum 33
Tm1 → Dm3v → TmY9q
max 4, sum 31
Mi4 → TmY10 → TmY9q
max 8, sum 55
Mi1 → T4a → TmY9q
max 15, sum 49
L3 → Tm20 → TmY9q
max 1, sum 7
Tm1 → TmY9q → Dm3p → TmY9q
